# Preoperative transferrin level is a novel prognostic marker for colorectal cancer

**DOI:** 10.1002/ags3.12411

**Published:** 2021-01-25

**Authors:** Hiroshi Sawayama, Yuji Miyamoto, Yukiharu Hiyoshi, Mototsugu Shimokawa, Rikako Kato, Takahiko Akiyama, Yuki Sakamoto, Nobuya Daitoku, Naoya Yoshida, Hideo Baba

**Affiliations:** ^1^ Department of Gastroenterological Surgery Graduate School of Medical Sciences Kumamoto University Kumamoto Japan; ^2^ Department of Biostatistics Graduate School of Medicine Yamaguchi University Yamaguchi Japan

**Keywords:** colorectal cancer, prognosis, transferrin

## Abstract

**Aim:**

This study investigated whether preoperative serum transferrin, a rapid‐turnover protein, was associated with prognosis after colorectal cancer (CRC) resection.

**Methods:**

We evaluated preoperative transferrin, which was calculated as iron and unsaturated iron‐binding capacity, in 501 patients who underwent surgery for Stage I–III CRC. Transferrin level was directly proportional to total iron‐binding capacity (TIBC), and TIBC < 250 μg/dl was defined as low transferrin. The associations between transferrin and prognosis were evaluated in univariate and multivariate Cox proportional hazards analyses.

**Results:**

Fifty‐eight of 501 patients (11.5%) had low transferrin. In these patients, low transferrin was significantly associated with high age, female gender, low body mass index (<18.5), high white blood cell count, low total protein, low albumin, high C‐reactive protein, low hemoglobin, and low neutrophil/lymphocyte ratio. In the univariate analysis, low transferrin was associated with shorter relapse‐free survival (RFS) (hazard ratio [HR] 2.180, 95% confidence interval [CI] 1.417‐3.354, *P* < .001), overall survival (OS) (HR 2.930, 95% CI 1.784‐4.811, *P* < .001), and cancer‐specific survival (CSS) (HR 2.122, 95% CI 1.053‐4.275, *P* = .035). In multivariate analysis, high age (*P* < .001), Glasgow Prognostic Score (*P* = .009), and low transferrin (HR 2.336, 95% CI 1.173‐4.654, *P* = .011) were independently associated with shorter OS, and depth of invasion pT4 (*P* = .015), presence of lymph node metastasis (*P* = .001), low hemoglobin (*P* = .034), and low transferrin (HR 2.638, 95% CI 1.113‐5.043, *P* = .025) were independently associated with shorter CSS.

**Conclusions:**

Preoperative serum transferrin in Stage I–III CRC patients was identified as a novel prognostic marker by univariate and multivariate analyses.

## Graphical abstract

Fifty‐eight of 501 patients CRC patients (11.5%) had low transferrin, which is a rapid turnover protein. Preoperarive low transferrin was associated with shorter relapse‐free survival (*P* < .001), overall survival (*P* < .001), and cancer‐specific survival (*P* = .004) compared with normal transferrin. Preoperative serum transferrin was identified as a novel prognostic marker in univariate and multivariate analyses.

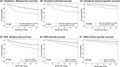

## 1 INTRODUCTION

Colorectal cancer (CRC) is the third most commonly diagnosed malignant disease in men and the second in women worldwide.^1^ The most effective treatment for resectable CRC is surgical resection with lymph node (LN) dissection and adjuvant chemotherapy after surgery in Stage III patients,^2^ although many patients, even in this group, suffered from recurrence or cancer‐specific mortality after curative surgery and adjuvant chemotherapy.

Prognostic factors have been analyzed to predict CRC patient survival, to investigate novel perioperative strategies, and follow‐up with the aim of improving CRC patients’ survival. Inflammatory and nutritional parameters were associated with patient prognosis in various cancers.^3^, ^4^ Many inflammatory and nutritional parameters have been analyzed and scoring systems were constructed to estimate the prognosis in CRC patients.^5^ C‐reactive protein (CRP), albumin, lymphocytes, and cholesterol are measured in usual clinical practice, and these parameters have been evaluated using the Glasgow Prognostic Score, which consists of CRP and albumin and predicts the survival of CRC patients^6^; nutritional scoring systems, prognostic nutritional index,^7^ and controlling nutritional status^8^ have also been associated with prognosis for cancer patients.

Transferrin, prealbumin, and retinal‐binding protein are rapid‐turnover proteins. The half‐life of albumin is 21 days, but the half‐lives of transferrin, prealbumin, and retinal‐binding protein are 7 days, 1.9 days, and 12 hours, respectively ^9^. These parameters were measured after surgery to estimate real‐time nutritional status,^10^ but because they were not usually measured before surgery, the association between preoperative values for these parameters and the short‐term or long‐term outcome of CRC patients who underwent surgery remains unclear. On the other hand, transferrin level before surgery can be estimated, because transferrin is directly proportional to total iron‐binding capacity.^9^

The aim of this study was to evaluate the relationship between preoperative serum transferrin status and the short‐term and long‐term outcomes in Stage I–III CRC patients who underwent colorectal resection. The results provide a novel insight into the association between preoperative transferrin status and prognosis of CRC patients, and we propose that transferrin should be measured to evaluate preoperative nutritional status and the need for nutritional support.

## 2 MATERIALS AND METHODS

### 2.1 Patients and evaluation of transferrin status

Between January 2005 and March 2018, 687 Stage I–III CRC patients underwent surgery at the Department of Gastroenterological Surgery, Kumamoto University. Of these 687 patients, blood iron and unsaturated iron‐binding capacity were measured in 501 patients, among whom 174 (34.7%) had Stage I CRC, 181 (36.1%) had Stage II CRC, and 146 (29.1%) had stage III CRC. Tumors were staged following the Japanese Classification of Colorectal Cancer.^11^ Transferrin was estimated by adding the value of iron to unsaturated iron‐binding capacity.^12^ The lower limit of the normal range of total iron‐binding capacity (TIBC) was 250 μg/dl, and TIBC < 250 μg/dl was therefore defined as low transferrin. Clinical data, including age, gender, body mass index, depth of invasion (pT), presence of metastatic LN (pN), pathological type, lymphatic invasion, and vascular invasion, were retrospectively available for 501 patients. Laboratory measurements included carcinoembryonic antigen (CEA), carbohydrate antigen 19‐9 (CA19‐9), white blood cells (WBC), serum total protein, albumin, CRP, hemoglobin, platelets, peripheral neutrophils, and lymphocytes. Each cut‐off value was defined based on the recommendations of the measuring kits our institute adopted. The cut‐off values of hemoglobin in males and females were 13.0 and 12.0 g/dl, respectively, which were defined based on the World Health Organization’s definitions. Neutrophil‐to‐lymphocyte ratio (NLR) and platelet‐to‐lymphocyte ratio (PLR), as inflammatory markers, were evaluated because the association between these markers and metastatic CRC patient prognosis has been reported. Cut‐off values of NLR and PLR were determined as 5 and 150, respectively, in accordance with previous studies.^5^ Glasgow prognostic score (GPS) was defined based on the presence of hypoalbuminemia (<35 g/l) and elevated CRP (>10 mg/l): if both were abnormal, the score was 2; if either one or the other were abnormal, the score was 1; if neither were abnormal, the score was 0 in accordance with a previous report.^13^ Complication after surgery was classified in accordance with the Clavien‐Dindo classification.^14^ Written informed consent was obtained from all the patients for the treatments.

### 2.2 Treatment strategy and follow‐up evaluation

The treatment strategy followed the Japanese colorectal cancer guidelines,^2^ which recommend surgery with LN dissection for Stage I–III CRC. Patients were followed up at 3‐month intervals. Recurrence was confirmed by clinical examinations, including computed tomography (CT). Tumor marker levels were measured every 3 months for 5 years after surgery. CT scanning studies that included the neck to the pelvis were performed at least twice a year for 3 years after surgery.

### 2.3 Statistical analysis

The association of transferrin with recorded clinical and pathological characteristics was determined by chi‐squared and Fisher's exact tests. All *P* values were two‐sided; *P* < .05 was considered significant. Analysis of risk factors for survival included age, gender, depth of invasion, LN metastasis, hemoglobin, GPS, NLR, PLR, and transferrin. Mortality was estimated from relapse‐free survival (RFS), overall survival (OS), and cancer‐specific survival (CSS). The log‐rank test was used in the survival analysis; the Kaplan‐Meier method was used to assess cumulative survival. Cox proportional hazards regression models were utilized to calculate hazard ratio (HR) and 95% confidence interval (CI). We performed multivariate Cox proportional hazards regression analysis to compute a HR according to age ≥ 70, male sex, depth of invasion (pT4), presence of LN metastasis, GPS, low hemoglobin, NLR (≥5), PLR (≥150), and transferrin. Backward stepwise elimination (likelihood method) with a threshold of *P* = .20 was used to select variables for the final model. Prognostic analysis was performed following the REMARK Guidelines.^15^ All data were processed and analyzed using the PASW Statistics 18 software program.

## 3 RESULTS

### 3.1 Association between transferrin and clinicopathological findings

Fifty‐eight of 501 patients (11.6%) had low transferrin. For these patients, low transferrin was significantly associated with high age (≥70) (*P* = .0298), female gender (*P* = .0219), low body mass index (<18.5) (*P* = .0269), high WBC (≥8600) (*P* = .0141), low total protein (*P* < .0001), low albumin (*P* < .0001), high CRP (*P* = .0119), low hemoglobin (*P* < .0001), and high NLR (*P* = .0047) in univariate analysis (Table 1).

**Table 1:** Association between serum transferrin level and clinicopathological factors

Factors	Total N = 501	Low transferrin N = 58	Normal transferrin N = 443	*P*‐value	
Age (years old)					
<70	257	22(37.9%)	235(53.0%)	.0298	*
≥70	244	36(62.1%)	208(47.0%)		
Gender					
Male	225	18(31.0%)	207(46.7%)	.0219	*
Female	276	40(69.0%)	236(53.3%)		
BMI (kg/m^2^)					
<18.5	58	15(25.9%)	43(9.7%)	.0269	*
18.5‐25	327	35(60.3%)	292(65.9%)		
25<	116	8(13.8%)	108(24.4%)		
Tumor location					
right‐sided	162	25(43.1%)	137(30.9%)	.0677	
left‐sided	339	33(56.9%)	306(69.1%)		
Depth of invasion					
pT1‐3	424	44(75.9%)	380(85.8%)	.0624	
pT4	77	14(24.1%)	63(14.2%)		
LN metastasis					
Absent	355	43(74.1%)	312(69.5%)	.5590	
Present	146	15(25.9%)	131(30.5%)		
Pathological type					
tub, pap	470	53(91.4%)	417(94.1%)	.4350	
por, sig, muc	31	5(8.6%)	26(5.9%)		
Lymphatic invasion					
Absent	398	48(82.8%)	350(79.0%)	.4590	
Present	103	10(17.2%)	93(21.0%)		
Vascular invasion					
Absent	263	30(51.7%)	233(52.6%)	.9005	
Present	238	28(48.3%)	210(47.4%)		
CEA (ng/ml)					
<3.4	262	25(43.1%)	237(53.5%)	.1361	
≥3.4	239	33(56.9%)	206(46.5%)		
CA19‐9 (U/ml)					
<37	425	48(82.8%)	377(85.1%)	.6451	
≥37	76	10(17.2%)	66(14.9%)		
WBC (/μl)					
<8600	442	45(77.6%)	397(89.6%)	.0141	*
≥8600	59	13(22.4%)	46(10.4%)		
Total protein (g/dl)					
<6.6	200	38(65.5%)	162(36.6%)	<.0001	*
≥6.6	301	20(34.5%)	281(63.4%)		
Albumin (g/dl)					
<4.1	324	55(94.8%)	269(60.7%)	<.0001	*
≥4.1	177	3(5.2%)	174(39.3%)		
CRP (mg/dl)					
<0.14	250	20(34.5%)	230(51.9%)	.0119	*
≥0.14	251	38(65.5%)	213(48.1%)		
Hemoglobin (g/dl)					
Male:<13, Female:<12	265	46(79.3%)	219(49.4%)	<.0001	*
Male:≥13, Female:≥12	236	12(20.7%)	224(50.6%)		
Neutrophil/lymphocyte					
<5	460	47(81.0%)	413(93.2%)	.0047	*
≥5	41	11(19.0%)	30(6.8%)		
Platelets/lymphocyte					
<150	268	32(55.2%)	236(53.3%)	.7849	
≥150	233	26(44.8%)	207(46.7%)		

Abbreviations: BMI, Body mass index; CA19‐9, carbohydrate antigen 19‐9; CEA, Carcinoembryonic antigen; CRP, C‐reactive protein; LN, lymph node; WBC, white blood cell.

* Significant difference.

### 3.2 Association between transferrin and survival

**Figure 1:**
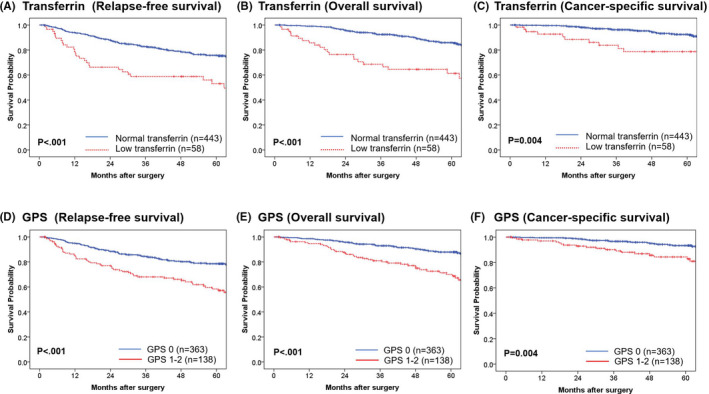
Kaplan‐Meier curves of relapse‐free survival (A), overall survival (B), and cancer‐specific survival (C) of colorectal cancer patients who underwent colorectal resection, in relation to their preoperative serum transferrin level. Kaplan‐Meier curves of relapse‐free survival (D), overall survival (E), and cancer‐specific survival (F) of colorectal cancer patients who underwent colorectal resection, in relation to their preoperative GPS score. GPS: Glasgow Prognostic Score

A total of 501 patients, of whom 78 (15.6%) had recurrence, 87 (17.4%) were dead, and 46 (9.1%) were cancer‐specific deaths, were monitored over a median follow‐up of 53.4 months. Five‐year RFS, OS, and CCS rates were 73.1%, 82.8%, and 91.0%. We evaluated the association between survival and transferrin status with the Kaplan‐Meier method. Low transferrin was significantly associated with shorter RFS (*P* < .001), OS (*P* < .001), and CSS (*P* = .004) compared with normal transferrin. Five‐year RFS rates of CRC patients with low and normal transferrin were 53.0% and 75.7%, respectively. Five‐year OS rates of CRC patients with low and normal transferrin were 61.2% and 85.9%, respectively. Five‐year CSS rates of CRC patients with low and normal transferrin were 78.7% and 92.5%, respectively (Figure 1A–C).

### 3.3 Association between GPS and survival

GPS was reported as a prognostic factor after colorectal surgery. In our study, the CRC patients with preoperative GPS 1 and GPS 2 had shorter PFS (*P* < .001), OS (*P* < .001), and CSS (*P* = .014) than those with preoperative GPS 0 (Supplementary Figure S1A‐C). Because there was no difference between the prognoses of patients with GPS 1 and GPS 2, we divided the 501 patients into two group, GPS 0 and GPS 1‐2. GPS 1‐2 was associated with unfavorable prognosis compared with GPS 0 (PFS (*P* < .001), OS (*P* < .001), and CSS (*P* = .004) (Figure 1D–F).

### 3.4 Association between clinicopathological factors and survival

Many clinicopathological factors were associated with RFS, OS, and CSS after colorectal resection. Low transferrin was also significantly associated with RFS (hazard ratio [HR] 2.180, 95% confidence interval [CI)] 1.417‐3.354, *P* < .001), OS (HR 2.930, 95% CI 1.784‐4.811, *P* < .001), and CSS (HR 2.122, 95% CI 1.053‐4.275, *P* = .035) in univariate Cox proportional hazards regression analysis. Low transferrin was strongly associated with unfavorable prognosis compared with normal transferrin in Stage III CRC patients. Low transferrin was associated with shorter RFS and OS in Stage I CRC patients. Transferrin level was not associated with survival in Stage II patients (Table 2).

**Table 2:** Prognosis of CRC patients in accordance with Stage

Factors	Ref.	Number	Relapse‐free survival			Overall survival			Cancer‐specific survival		
			HR	95% CI	*P*‐value	HR	95% CI	*P*‐value	HR	95% CI	*P*‐value
Transferrin: low	Normal	501	2.180	(1.417‐3.354)	<.001*	2.930	(1.784‐4.811)	<.001*	2.122	(1.053‐4.275)	.035*
Stage I		174	3.790	(1.600‐8.126)	.003*	4.026	(1.713‐8.844)	.002*	2.818	(0.616‐9.779)	.162
Stage II		181	1.218	(1.496‐2.564)	.640	1.345	(0.449‐3.273)	.564	0.634	(0.035‐3.240)	.634
Stage III		146	4.447	(2.089‐8.560)	<.001*	8.893	(3.394‐20.86)	<.001*	12.09	(4.125‐31.87)	<.001*

Abbreviations: CI, confidence interval; HR, hazard ratio; Ref, reference.

* Significant difference.

High age (*P* = .012), depth of invasion pT4 (*P* = .013), presence of LN metastasis (*P* < .001), GPS 1‐2 (*P* = .046), and low transferrin were significantly associated with RFS in multivariate Cox proportional hazards regression analysis (low transferrin: HR 1.797, 95% CI 1.124‐2.871, *P* = .014) (supplementary Table S1, supplementary Figures S2 and S5). High age (*P* < .001), GPS 1‐2 (*P* = .009), and low transferrin were significantly associated with OS in multivariate Cox proportional hazards regression analysis (low transferrin: HR 2.336, 95% CI 1.173‐4.654, *P* = .011) (Table 3, supplementary Figures S3 and S6). Depth of invasion pT4 (*P* = .015), presence of LN metastasis (*P* = .001), low hemoglobin (*P* = .034), and low transferrin were independently associated with CSS in multivariate Cox proportional hazards regression analysis (low transferrin: HR 2.369, 95% CI 1.113‐5.043, *P* = .025) (Table 4, supplementary Figures S4 and S7).

**Table 3:** Overall survival of CRC patients in univariate and multivariate Cox proportional hazards analysis

Factors	Ref.	Univariate analysis				Multivariate analysis			
		HR	95% CI	*P*‐value		HR	95% CI	*P*‐value	
Age: ≥70	<70	2.313	(1.480‐3614)	<.001	*	2.356	(1.486‐3.737)	<.001	*
Gender: Male	Female	1.035	(0.674‐1.590)	.874					
Depth of invasion: pT4	pT1‐3	1.978	(1.221‐3.207)	.006	*	1.423	(0.841‐2.407)	.188	
LN metastasis: Present	Absent	1.472	(0.949‐2.285)	.084		1.553	(0.985‐2.449)	.058	
Hemoglobin (g/dl): Low ^a^	Normal	2.492	(1.565‐3.958)	<.001	*	1.608	(0.972‐2.662)	.064	
Glasgow prognostic score: 1 or 2	0	2.729	(1.788‐4.167)	<.001	*	1.902	(1.175‐3.080)	.009	*
Neutrophil/lymphocyte: ≥5	<5	1.346	(0.650‐2.787)	.424					
Platelets/lymphocyte: ≥150	<150	1.014	(0.664‐1.548)	.949					
Transferrin: Low	Normal	2.930	(1.784‐4.811)	<.001	*	2.336	(1.173‐4.654)	.011	*

Abbreviations: CI, confidence interval; HR, hazard ratio; LN, lymph node.

^a^ Low: Male: <13 g/dL, Female: <12 g/dL, Normal: Male: ≥13 g/dL, Female: ≥12 g/dL.

* Significant difference.

**Table 4:** Cancer‐specific survival of CRC patients in univariate and multivariate Cox proportional hazards analysis

Factors	Ref.	Univariate analysis				Multivariate analysis			
		HR	95% CI	*P*‐value		HR	95% CI	*P*‐value	
Age: ≥70	<70	1.629	(0.904‐2.933)	.104		1.589	(0.868‐2.912)	.134	
Gender: Male	Female	0.789	(0.441‐1.412)	.425					
Depth of invasion: pT4	pT1‐3	3.113	(1.693‐5.724)	<.001	*	2.260	(1.177‐4.341)	.015	*
LN metastasis: Present	Absent	2.644	(1.483‐4.715)	.001	*	2.750	(1.495‐5.057)	.001	*
Hemoglobin (g/dl): Low ^a^	Normal	2.582	(1.358‐4.910)	.004	*	2.062	(1.057‐4.024)	.034	*
Glasgow prognostic score: 1 or 2	0	2.321	(1.295‐4.161)	.005	*				
Neutrophil/lymphocyte: ≥5	<5	1.240	(0.444‐3.460)	.681					
Platelets/lymphocyte: ≥150	<150	0.964	(0.538‐1.728)	.902					
Transferrin: Low	Normal	2.122	(1.053‐4.275)	.035	*	2.369	(1.113‐5.043)	.025	*

Abbreviations: CI, confidence interval; HR, hazard ratio; LN, lymph node.

^a^ Low: Male: <13 g/dL, Female: <12 g/dL, Normal: Male: ≥13 g/dL, Female: ≥12 g/dL.

* Significant difference.

### 3.5 Association of transferrin with blood transfusion and postoperative complication

**Figure 2:**
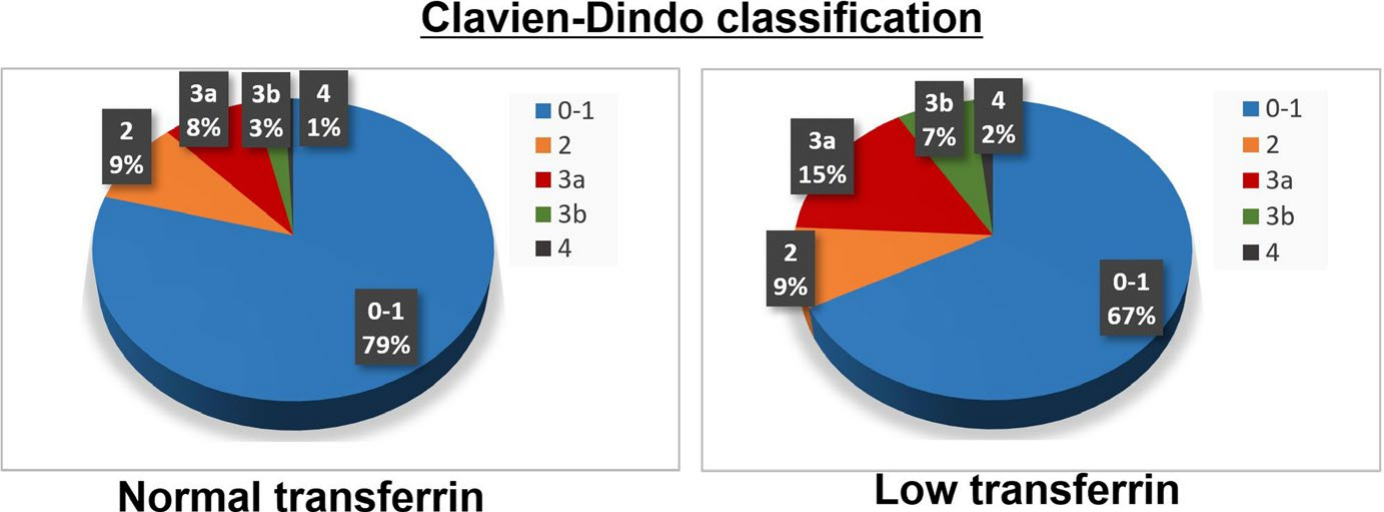
The association between postoperative complication and preoperative transferrin, in accordance with the Clavien‐Dindo classification

The association of the prognosis of CRC patients after surgery with blood transfusion and postoperative complication was previously reported.^16^, ^17^ Preoperative and intraoperative blood transfusions were more frequently performed for patients with low transferrin compared with those with normal transferrin (*P* = .0124 and 0.0063, respectively). Postoperative complication was evaluated with the Clavien‐Dindo classification. The transferrin level was not significantly associated with anastomotic leakage (*P* = .4609); however, patients with low transferrin tended to suffer from postoperative complication with Clavien‐Dindo classification ≥ 3 compared to those with normal transferrin (*P* = .0696) (Table 5 and Figure 2).

**Table 5:** Association between transferrin and blood transfusion and postoperative complications

Factors	Total	Low Transferrin	Normal Transferrin	*P*‐value	
	N = 501	N = 58	N = 443		
Preoperative blood transfusion					
Not done	478	51 (87.9%)	427 (96.4%)	.0124	*
Done	23	7 (12.1%)	16 (3.6%)		
Intraoperative blood transfusion					
Not done	434	43 (74.1%)	391 (88.3%)	.0063	*
Done	67	15 (25.9%)	52 (11.7%)		
Anastomotic leakage					
Absent	474	56 (96.6%)	418 (94.4%)	.4609	
present	27	2 (3.4%)	25 (5.6%)		
Postoperative complication					
Clavien–Dindo 0‐2	423	44 (75.9%)	379 (85.6%)	.0696	
Clavien–Dindo 3‐4	78	14 (24.1%)	64 (14.4%)		

* Significant difference.

## 4 DISCUSSION

Preoperative transferrin levels were significantly associated with RFS, OS, and CSS of Stage I–III CRC patients in univariate and multivariate analyses. Preoperative low serum transferrin in CRC patients undergoing surgery was detected as a novel prognostic marker, and further research on this finding will be required.

Because inflammation and nutrition are deeply associated with CRC patient survival, we evaluated the association between inflammation and/or nutrition status and patient prognosis. Elevated preoperative CRP was associated with prognosis of Stage I–III CRC patients in a meta‐analysis of 21 studies, including a total of 3934 CRC patients. The cut‐off value for CRP was 0.5 or 1 mg/dl. CRC patients with elevated CRP had shorter OS (HR 2.04, 95% CI 1.45‐2.86) and shorter CSS (HR 4.37, 95% CI 2.63‐7.27) than patients whose CRP was not elevated ^18^. Lai et al reported that hypoalbuminemia is a predictor of poor prognosis for CRC patients. In their study, 2529 patients were analyzed for long‐term outcome. Five‐year relapse‐free survival rates of hypoalbuminemia and normal albumin patients were 78.9% and 73.5%, respectively (HR 1.28, 95% CI 1.04‐1.56, *P* = .020), and five‐year OS rates were 78.0% and 60.0%, respectively (HR 1.75, 95% CI 1.49‐2.08, *P* < .0001). The study indicated that albumin was strongly associated with OS.^19^ CRP and albumin were prognostic factors for CRC patients in another study.^20^ Several scoring systems have shown that these inflammatory and nutritional factors are associated with CRC patient prognosis. One of these, the Glasgow Prognostic Score (GPS), is based on the combination of CRP and serum albumin levels. CRC patients with elevated GPS or modified GPS were significantly associated with shorter OS (HR 2.20, 95% CI 1.88‐2.57, *P* < .001) and shorter CSS (HR 1.86, 95% CI 1.59‐2.17, *P* < .001) in a meta‐analysis.^21^ In our study, GPS was evaluated as an inflammatory and nutritional marker in multivariate analysis, because GPS includes albumin and CRP. GPS was associated with RFS and OS, although it was not independently associated with CSS. However, low transferrin was independently associated with shorter CSS as well as shorter RFS and OS in multivariate analysis.

Transferrin is a rapid‐turnover protein and is synthesized in the liver. It is one of the nutrition markers. A preoperative immunonutrition pharmaceutics diet and prebiotics in patients with gastrointestinal cancer increased immunoglobulin as well as transferrin. Nutritional status may be tightly associated with immunity. Improving nutritional status and immunity reduce the incidence of postoperative complications and infections.^22^, ^23^ The preoperative serum transferrin level is a possible predictive marker of postoperative pneumonia after esophageal surgery.^24^ Serum transferrin was also predictive of spontaneous closure in patients with gastrointestinal cutaneous fistulas.^25^ These reports suggested that transferrin may be a surrogate marker of immunity and wound healing as well as nutritional status. Furthermore, the IL‐6, IL‐8, VEGF‐A, and midkine cytokines were elevated in cachectic patients in gastroesophageal cancers. Transferrin was decreased in these cachectic patients.^26^, ^27^ Adjuvant chemotherapy improves the prognosis of Stage III CRC patients. In this study, adjuvant chemotherapy was performed for 101 of the 146 Stage III CRC patients (68.2%). The number of Stage III CRC patients with normal transferrin was 131, and 97 of these (74.0%) received adjuvant chemotherapy; in contrast, 15 Stage III CRC patients had low transferrin, and four of them (26.6%) underwent adjuvant chemotherapy. Low transferrin patients thus rarely receive adjuvant chemotherapy (*P* < .001) and this may be one of the reasons that low transferrin was associated with unfavorable prognosis in Stage III patients. An association between transferrin and CRC patients’ prognosis was therefore uncovered in our study, and further investigation and analysis are needed to reveal the mechanism of how transferrin affects prognosis.

Transferrin level is reportedly decreased by surgical stress but recovers after surgery. Nutritional support has been tried for patients with upper gastrointestinal cancer,^28^, ^29^ because their oral intake decreases after gastrectomy and esophagectomy.^30^, ^31^ However, most CRC patients can eat after surgery, and nutritional support after surgery has not been discussed. In this study, about 10% of the CRC patients displayed low transferrin. These patients were more likely to have both postoperative complications and worse long‐term outcomes than patients with normal transferrin. Not all CRC patients are likely to need aggressive nutritional support. However, those with low transferrin may need perioperative nutritional support, and transferrin, a rapid‐turnover protein, may be an optimal index to estimate real‐time nutritional status.

This study had limitations. It was a retrospective study and 186 of the 687 CRC patients (27.1%) were not evaluated for their transferrin level before surgery. This potentially leads to selection bias. The nutritional support provided depended on the attending doctor, because the doctors had not participated in any trials of immune nutrition support.

In conclusion, preoperative serum transferrin, a rapid‐turnover protein, in patients who underwent CRC resection was identified as a prognostic marker by univariate and multivariate analysis. Aggressive perioperative nourishment support for patients with low transferrin may improve the prognosis of patients who receive CRC resection.

## CONFLICT OF INTEREST

The authors declare no conflict of interests for this article.

## Supporting information

Fig S1

Fig S2

Fig S3

Fig S4

Fig S5

Fig S6

Fig S7

Table S1
