# Supplementary material for: Preoperative transferrin level is a novel prognostic marker for colorectal cancer
Source: Ann Gastroenterol Surg. 2021 Jan 25;5(2):243–51. doi: 10.1002/ags3.12411 (PMC8034684; doi:10.1002/ags3.12411)
Supplement: Supplementary file 1 — Fig S1 [file AGS3-5-243-s002.pptx]

## Slide 1
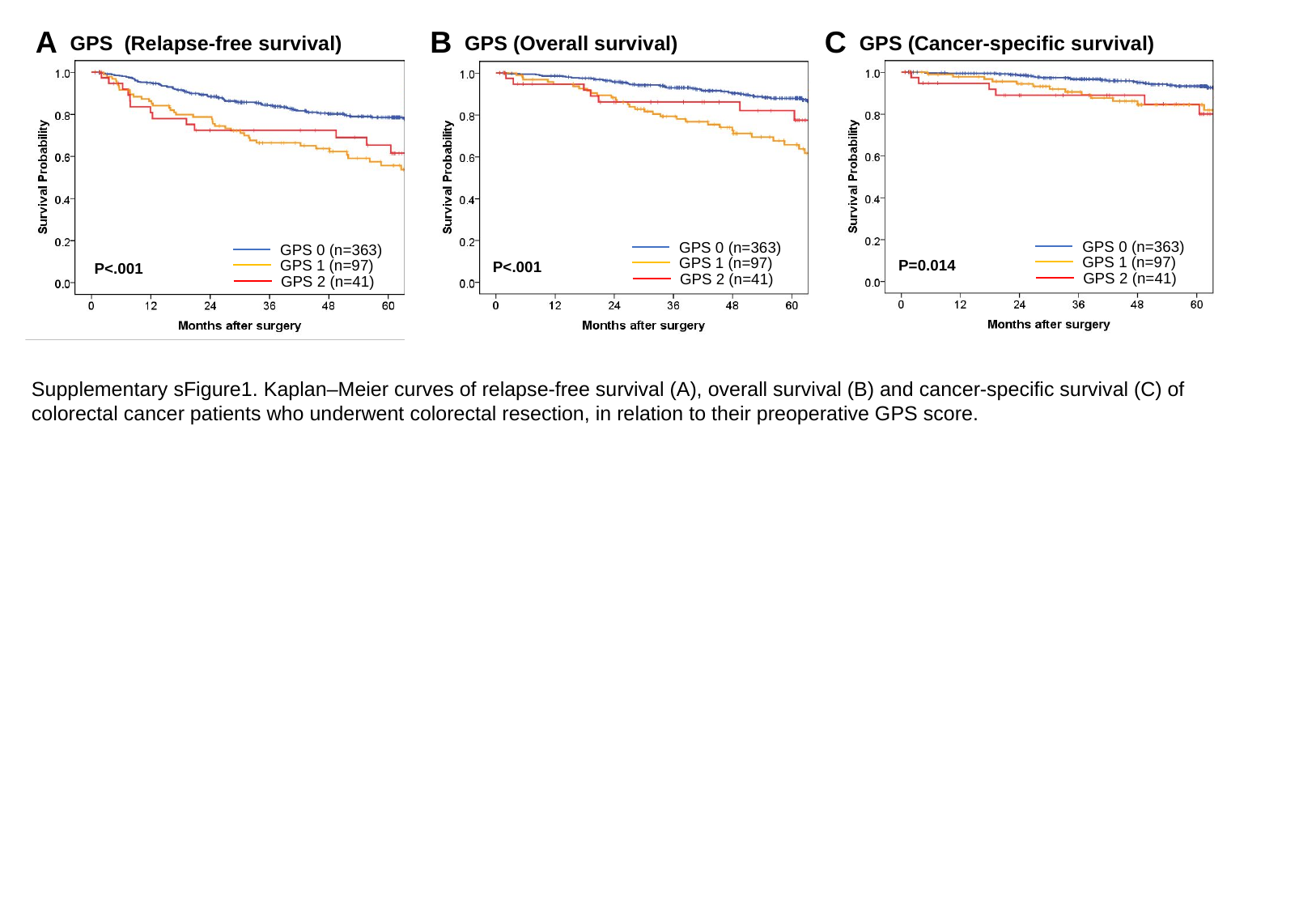

A
B
C
GPS (Relapse-free survival)
GPS (Overall survival)
GPS (Cancer-specific survival)
GPS 0 (n=363)
GPS 0 (n=363)
GPS 0 (n=363)
GPS 1 (n=97)
GPS 1 (n=97)
P=0.014
GPS 1 (n=97)
P<.001
P<.001
GPS 2 (n=41)
GPS 2 (n=41)
GPS 2 (n=41)
Supplementary sFigure1. Kaplan–Meier curves of relapse-free survival (A), overall survival (B) and cancer-specific survival (C) of colorectal cancer patients who underwent colorectal resection, in relation to their preoperative GPS score.
