# Supplementary material for: Preoperative transferrin level is a novel prognostic marker for colorectal cancer
Source: Ann Gastroenterol Surg. 2021 Jan 25;5(2):243–51. doi: 10.1002/ags3.12411 (PMC8034684; doi:10.1002/ags3.12411)
Supplement: Supplementary file 3 — Fig S3 [file AGS3-5-243-s004.pptx]

## Slide 1
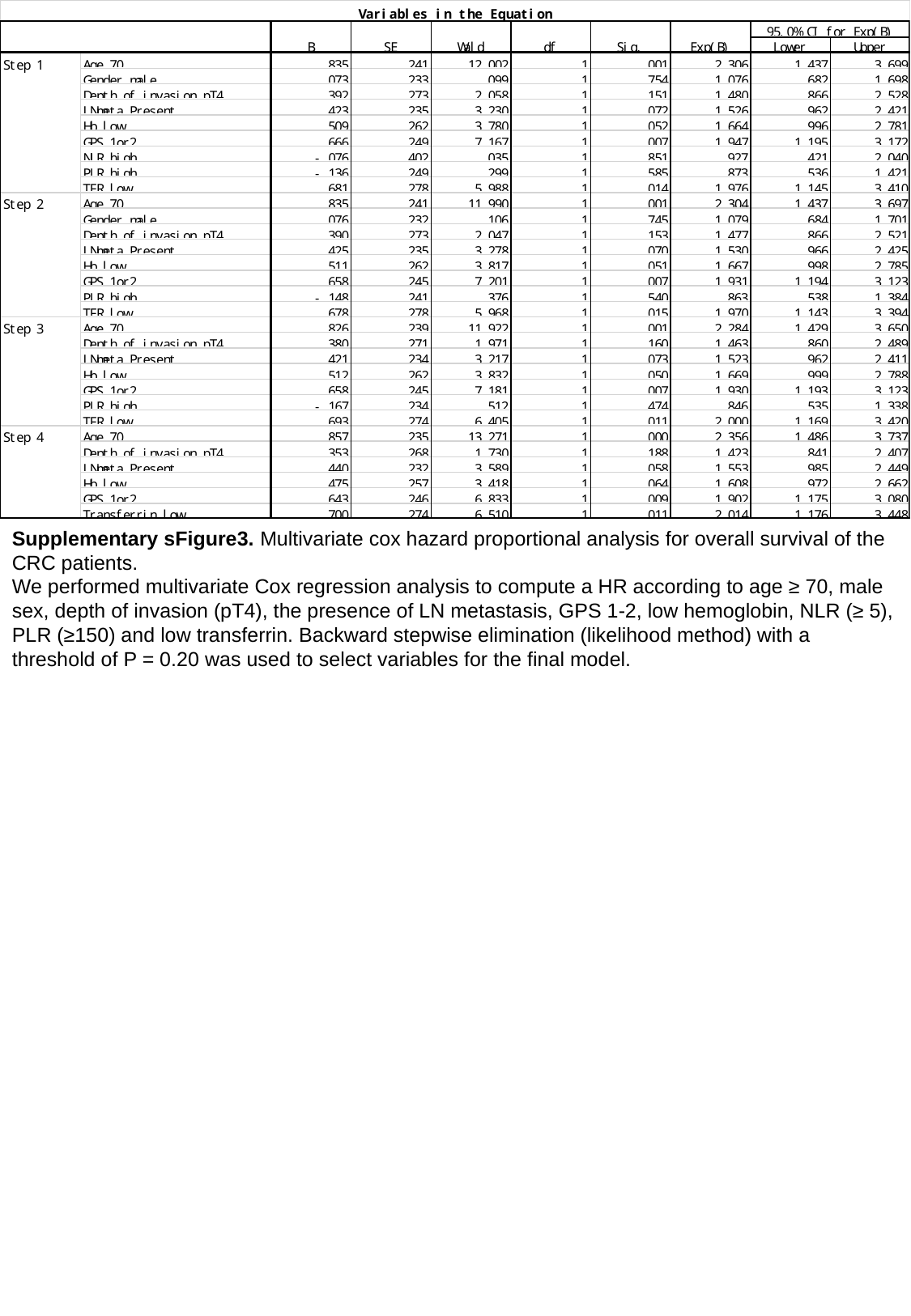

Supplementary sFigure3. Multivariate cox hazard proportional analysis for overall survival of the CRC patients.
We performed multivariate Cox regression analysis to compute a HR according to age ≥ 70, male sex, depth of invasion (pT4), the presence of LN metastasis, GPS 1-2, low hemoglobin, NLR (≥ 5), PLR (≥150) and low transferrin. Backward stepwise elimination (likelihood method) with a threshold of P = 0.20 was used to select variables for the final model.
