# Supplementary material for: Preoperative transferrin level is a novel prognostic marker for colorectal cancer
Source: Ann Gastroenterol Surg. 2021 Jan 25;5(2):243–51. doi: 10.1002/ags3.12411 (PMC8034684; doi:10.1002/ags3.12411)
Supplement: Supplementary file 5 — Fig S5 [file AGS3-5-243-s001.pptx]

## Slide 1
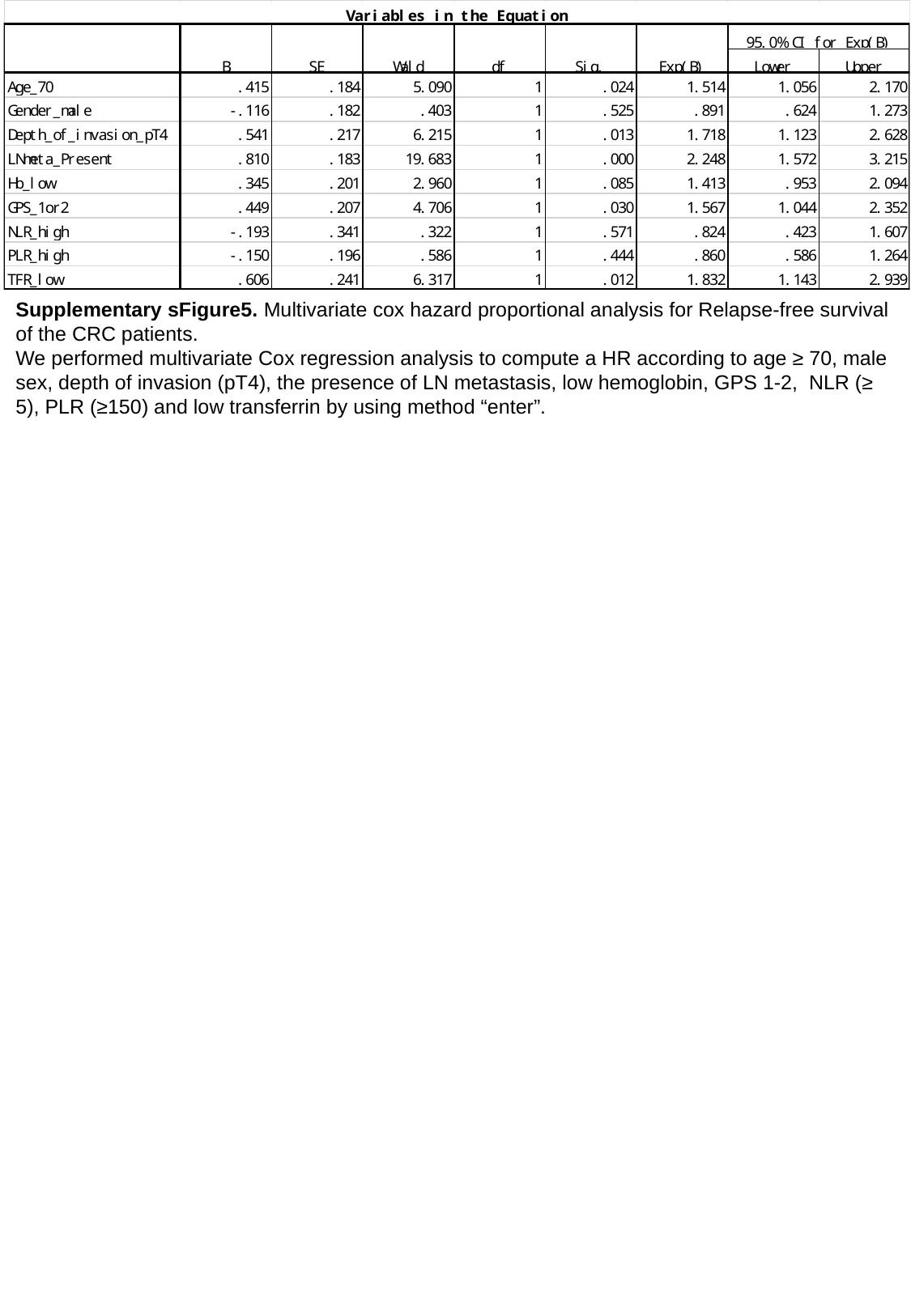

Supplementary sFigure5. Multivariate cox hazard proportional analysis for Relapse-free survival of the CRC patients.
We performed multivariate Cox regression analysis to compute a HR according to age ≥ 70, male sex, depth of invasion (pT4), the presence of LN metastasis, low hemoglobin, GPS 1-2, NLR (≥ 5), PLR (≥150) and low transferrin by using method “enter”.
