# Supplementary material for: Preoperative transferrin level is a novel prognostic marker for colorectal cancer
Source: Ann Gastroenterol Surg. 2021 Jan 25;5(2):243–51. doi: 10.1002/ags3.12411 (PMC8034684; doi:10.1002/ags3.12411)
Supplement: Supplementary file 8 — Table S1 [file AGS3-5-243-s007.docx]

**Supplementary sTable 1.** Relapse-free survival of CRC patients in univariate and multivariate Cox proportional hazards analysis.

|  |  |  | Univariate analysis | | |  | Multivariate analysis | | |  |
| --- | --- | --- | --- | --- | --- | --- | --- | --- | --- | --- |
|  | Factors | Ref. | HR | 95% CI | P-value |  | HR | 95% CI | P-value |  |
|  | Age: ≥70 | <70 | 1.548 | (1.096-2.185) | 0.013 |  | 1.573 | (1.105-2.140) | 0.012 | ^*^ |
|  | Gender: Male | Female | 0.813 | (0.578-1.144) | 0.813 |  |  |  |  |  |
|  | Depth of invasion: pT4 | pT1-3 | 2.342 | (1.592-3.447) | <.001 | ^*^ | 1.695 | (1.116-2.576) | 0.013 | ^*^ |
|  | LN metastasis: Present | Absent | 2.249 | (1.595-3.171) | <.001 | ^*^ | 2.297 | (1.611-3.276) | <.001 | ^*^ |
|  | Hemoglobin (g/dl): Low ^¶^ | Normal | 1.808 | (1.266-2.581) | 0.001 | ^*^ | 1.368 | (0.931-2.011) | 0.110 |  |
|  | Glasgow prognostic score: 1 or 2 | 0 | 2.166 | (1.532-3.063) | <.001 | ^*^ | 1.504 | (1.008-2.243) | 0.046 | ^*^ |
|  | Neutrophil/lymphocyte: ≥5 | <5 | 1.192 | (0.643-2.211) | 0.577 |  |  |  |  |  |
|  | Platelets/lymphocyte: ≥150 | <150 | 1.031 | (0.733-1.450) | 0.862 |  |  |  |  |  |
|  | Transferrin: Low | Normal | 2.180 | (1.417-3.354) | <.001 | ^*^ | 1.797 | (1.124-2.871) | 0.014 | ^*^ |

HR: hazard ratio, CI: confidence interval, LN: lymph node

^¶^: Low: Male: < 13 g/dl, Female: < 12 g/dl, Normal: Male: ≥ 13 g/dl, Female: ≥ 12 g/dl. * Significant difference.
